# Field performance of the malaria highly sensitive rapid diagnostic test in a setting of varying malaria transmission

**DOI:** 10.1186/s12936-019-2929-1

**Published:** 2019-08-27

**Authors:** Julia Mwesigwa, Hannah Slater, John Bradley, Binta Saidy, Fatima Ceesay, Charles Whittaker, Ballah Kandeh, Davis Nkwakamna, Chris Drakeley, Jean-Pierre Van Geertruyden, Teun Bousema, Jane Achan, Umberto D’Alessandro

**Affiliations:** 1 Medical Research Council Unit The Gambia at London School of Hygiene and Tropical Medicine, P.O. Box 273, Banjul, The Gambia; 2 Faculty of Medicine and Health Sciences, University of Antwerp, Antwerp, Belgium; 3 MRC Centre for Global Disease Analysis, Department of Infectious Disease Epidemiology, Imperial College London, London, Norfolk Place, London, W2 1P UK; 4 PATH, 2201 Westlake Avenue, Seattle, USA; 5 MRC Tropical Epidemiology Group, London School of Hygiene and Tropical Medicine, London, UK; 6 National Malaria Control Programme, Banjul, Gambia; 7 Department of Immunology and Infection, Faculty of Infectious Diseases and Tropical Medicine, London School of Hygiene and Tropical Medicine, London, UK; 8 Radboud Institute for Health Sciences, Radboud University Medical Center, Nijmegen, The Netherlands

**Keywords:** Highly sensitive rapid diagnostic test, Malaria, Mass testing and treatment, *Plasmodium falciparum*, Transmission areas

## Abstract

**Background:**

The Gambia has successfully reduced malaria transmission. The human reservoir of infection could further decrease if malaria-infected individuals could be identified by highly sensitive, field-based, diagnostic tools and then treated.

**Methods:**

A cross-sectional survey was done at the peak of the 2017 malaria season in 47 Gambian villages. From each village, 100 residents were randomly selected for finger-prick blood samples to detect *Plasmodium falciparum* infections using highly sensitive rapid diagnostic tests (HS-RDT) and PCR. The sensitivity and specificity of the HS-RDT were estimated (assuming PCR as the gold standard) across varying transmission intensities and in different age groups. A deterministic, age-structured, dynamic model of malaria transmission was used to estimate the impact of mass testing and treatment (MTAT) with HS-RDT in four different scenarios of malaria prevalence by PCR: 5, 15, 30, and 60%, and with seasonal transmission. The impact was compared both to MTAT with conventional RDT and mass drug administration (MDA).

**Results:**

Malaria prevalence by HS-RDT was 15% (570/3798; 95% CI 13.9–16.1). The HS-RDT sensitivity and specificity were 38.4% (191/497, 95% CI 34.2–42.71) and 88.5% (2922/3301; 95% CI 87.4–89.6), respectively. Sensitivity was the highest (50.9%, 95% CI 43.3–58.5%) in high prevalence villages (20–50% by PCR). The model predicted that in very low transmission areas (≤ 5%), three monthly rounds of MTAT with HS-RDT, starting towards the end of the dry season and testing 65 or 85% of the population for 2 consecutive years, would avert 62 or 78% of malaria cases (over 2 years), respectively. The effect of the intervention would be lower in a moderate transmission setting. In all settings, MDA would be superior to MTAT with HS-RDT which would be superior to MTAT with conventional RDT.

**Conclusion:**

The HS-RDT’s field sensitivity was modest and varied by transmission intensity. In low to very low transmission areas, three monthly rounds per year of MTAT with HS-RDT at 85% coverage for 2 consecutive years would reduce malaria prevalence to such low levels that additional strategies may achieve elimination. The model prediction would need to be confirmed by cluster-randomized trials.

## Background

After a decade of considerable success in malaria control, progress has stalled, with no further reduction of malaria cases between 2015 and 2017 [1]. The Gambia is one of the sub-Saharan African countries where the number of malaria cases has steadily declined due to the scale up of malaria control interventions [2, 3]. However, malaria transmission has not been interrupted, possibly because of the persistence of asymptomatic infections, some of them sub-microscopic (sub-patent), that occur in all endemic settings [4, 5]. In The Gambia, 60% of infections identified during the dry season (January–June) and 30% of those during the transmission season (July–December) were asymptomatic, with a third of these sub-patent [6]. As transmission declines, the proportion of sub-patent infections among infected and asymptomatic individuals may increase, although their contribution to transmission remains unclear [4, 7].

Strategies such as mass drug administration (MDA) or mass testing and treatment (MTAT) target the human reservoir of infection and may decrease malaria prevalence to such low levels that other strategies may achieve elimination. While MDA aims at administering a full anti-malarial treatment to the whole population, without screening for either symptoms or infections [8], MTAT would treat only positive individuals identified through mass testing, regardless of symptoms [9]. The low sensitivity of conventional rapid diagnostic tests (RDTs), its detection limit is approximately 100–200 parasites/µl and comparable to light microscopy [5, 10–13], remains a major challenge for the success of MTAT campaigns. In Burkina Faso, Ethiopia and Kenya, MTAT did not achieve significant or sustained reductions in malaria transmission in communities [14, 15] and school children [16, 17], partly because conventional RDTs would not detect low-density, sub-patent infections [18, 19].

Low-density infections can be identified by more sensitive molecular diagnostic methods that detect the parasites’ nucleic acid. Depending on the blood volume examined and the molecular target, molecular tests can detect infections with parasite densities as low as 0.02–0.1 parasites/µl [20]. However, this approach is complex, expensive, and not easily field deployable as it requires skilled staff and a central laboratory [18, 21].

The highly sensitive RDTs (HS-RDT) were developed for the field detection of low-density infections, with a detection limit of 40–125 pg/ml histidine-rich protein-2 (HRP2), tenfold lower than that of conventional SD Bioline Malaria Ag P.f RDT (800–1000 pg/ml) [11]. When compared to quantitative reverse-transcription PCR (q)RT-PCR, HS-RDT’s sensitivity was 44% in asymptomatic individuals in Myanmar (low transmission), 47% in malaria naïve individuals infected experimentally with blood stage *Plasmodium falciparum* parasites, and 84% in Ugandan asymptomatic children (high transmission); its specificity was greater than 96% [22]. In Myanmar, under laboratory conditions, HS-RDT’s sensitivity was 51% compared to the combined high-volume ultrasensitive qPCR and Quansys human malaria 4-plex enzyme-linked immunosorbent assay (ELISA) tests, and 54% when compared to only ultrasensitive qPCR. In the field, the HS-RDT sensitivity was 36% compared to ultra-sensitive qPCR; HS-RDTs had a twofold higher sensitivity than conventional RDTs and microscopy [18]. In Papua New Guinea, HS-RDT detected 51% of the *P. falciparum* infections detected by ultra-sensitive qPCR and 26% of *P. falciparum* infections detected by both standard qPCR and ultra-sensitive qPCR [19].

It is unclear what effect MTAT with HS-RDT would have on malaria transmission as a proportion of infected people would remain undetected and untreated. The objective of this study was to determine the field sensitivity and specificity of HS-RDT (reference test: var gene acidic terminal sequence (varATS) PCR. These values were used to predict the effect of MTAT campaigns with HS-RDT on malaria prevalence using a mathematical model.

## Methods

### Study design and participants

**Fig. 1:**
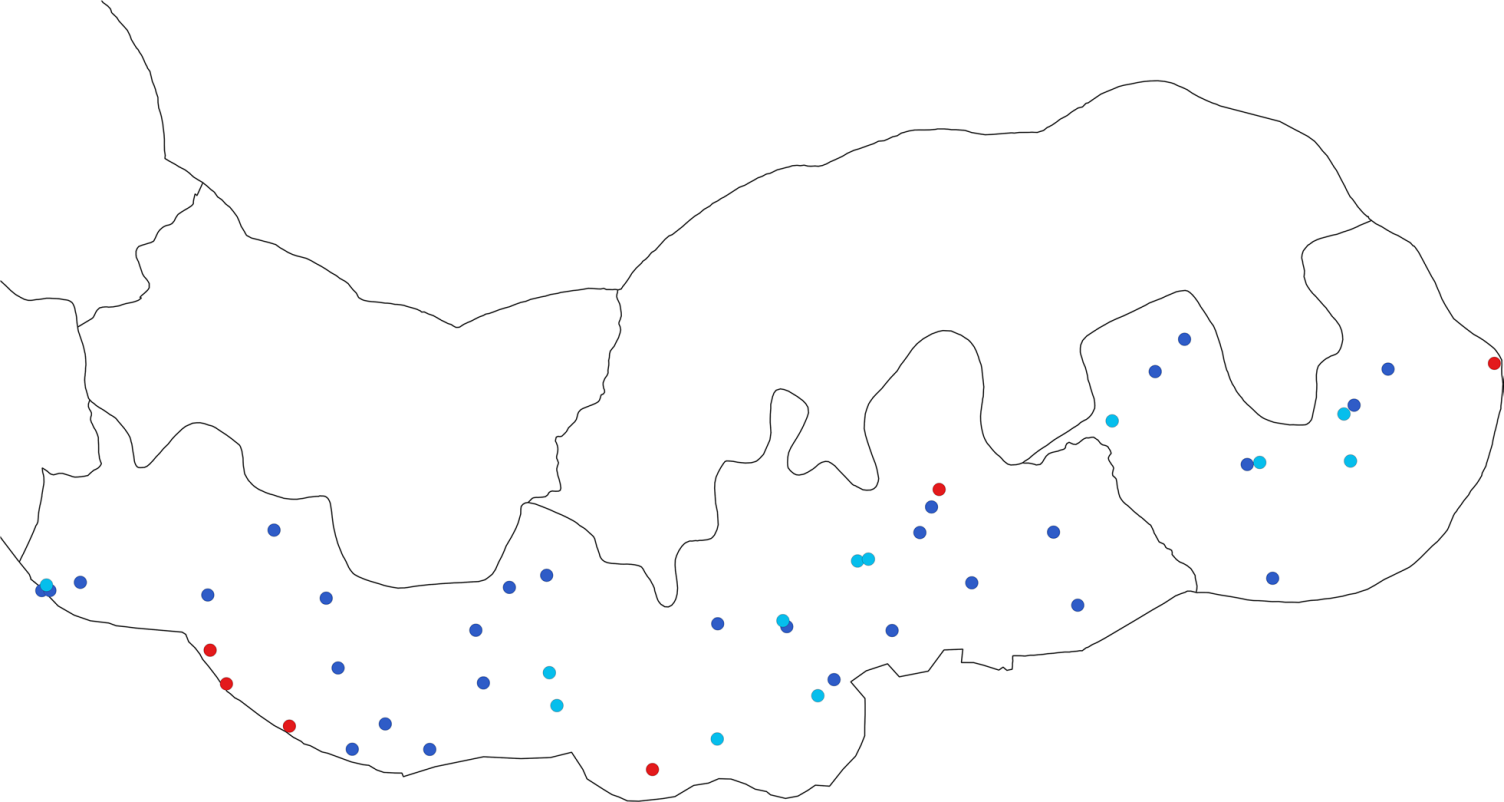
Study sites in upper river region-south bank and *Plasmodium falciparum* infection prevalence by PCR. Filled light blue circle: very low prevalence (< 5%), filled dark blue circle: low-moderate transmission (5 to < 20%), filled red circle: high prevalence (20–50%)

Malaria transmission in The Gambia is highly seasonal, with peak transmission from October to November. A cross-sectional survey was conducted over a month (7 November to 8 December 2017) in 47 villages in eastern Gambia. The selected villages with populations between 100 and 800 residents are included as part of the Health and Demographic Surveillance System (HDSS) (Fig. 1). In each village, individuals aged at least 6 months were randomly selected for the survey; in smaller villages, all the inhabitants were selected.

Individual finger-prick blood samples were collected for HS-RDT and diagnostic PCR, for the latter on filter paper to diagnose malaria infections. Selected participants underwent medical examination and axillary temperature was measured by a digital thermometer. Participants with a positive HS-RDT and fever (axillary temperature ≥ 37.5 °C) and/or history of fever in the previous 24 h were treated with artemether-lumefantrine, the first line treatment in The Gambia.

### Data management

Data was collected and managed using the REDCap (Research Electronic Data Capture) data capture tool that has an intuitive interface for validated data entry, audit trails for tracking and exporting data. Individual data were completed for demographic characteristics, history of fever and recent anti-malarial use, axillary temperature, long-lasting insecticidal net (LLIN) ownership and use, and prior indoor residual spraying (IRS).

### Sample size

Assuming malaria prevalence of 10%, 100 individuals per village would be able to estimate malaria prevalence with a precision of ± 6%.

### Malaria detection and laboratory analysis

The HS-RDT was carried out following the manufacturer’s recommendations. Briefly, a finger-prick blood sample was collected and delivered to the sample well in the test cassette. Four drops of buffer were added and the results were interpreted within 20 min by a study nurse [11].

The *var*ATS assay was used to generate qualitative *P. falciparum* results. Briefly, three 3-mm dried blood spots were punched into 96-well plates and digested in 20 µl of proteinase K and 180 µl of ATL tissue lysis buffer solution. The *Plasmodium* DNA was extracted using the QIAamp 96 DNA QIAcube HT kit (Qiagen, Germany) and Qiacube HT^®^ robot. Extracted DNA was eluted into 80 µl of elution buffer and stored at − 20 °C till further use. For the analysis, the *var* gene acidic terminal sequence (*var*ATS, 59 copies/genome) of *P. falciparum* was amplified [23]. All PCR reactions included 10 standards prepared from tenfold serially diluted samples containing known numbers of infected erythrocytes diluted in whole blood. The limit of detection of the PCR assay is approximately 0.2 parasites/µl of blood [23]. The PCR output was analysed using the BioRad CFX Manager software.

### Statistical analysis

Malaria prevalence was calculated by dividing the number of positive samples, either by HS-RDT or PCR, by the total number of tested samples. An asymptomatic infection was defined as an individual with positive HS-RDT or PCR without history of fever in the previous 24 h and axillary temperature ≤ 37.5 °C [24] at the time of blood collection. PCR was the reference test to estimate the diagnostic accuracy of HS-RDT. The sensitivity was estimated as follows: true positives/(true positives + false negatives); specificity as true negatives/(true negatives + false positives); positive predictive value as true positives/(true positive + false positives); negative predictive value as true negatives/(true negatives + false negatives). The Pearson’s Chi squared test was used to compare the HS-RDT’s sensitivity and specificity by age and transmission intensity. Univariate and multivariate logistic regression analysis was used to determine the odds of being “false negative” by age and the odds of being “false positive” by intensity of transmission. The regression models were adjusted for gender, IRS in the last 6 months and use of LLINs at night. Malaria prevalence by PCR was used as a proxy of transmission intensity, categorized as very low (< 5%,) low-moderate (5 to  < 20%) and high (20–50%) transmission. The association between the HS-RDT sensitivity and transmission intensity was determined by fitting a line between the two quantities derived from a log-odds regression model [4] implemented within a Bayesian based framework. This framework was implemented in the statistical software package JAGS, and parameter inference based on Markov Chain Monte Carlo simulations using the R package rjags version 4-8 (https://CRAN.R-project.org/package=rjags). Infection status was defined by four categories: (1) malaria-infected individuals with detectable HRP2 levels: both PCR and HS-RDT positives; (2) individuals with a recently cleared infection, either naturally or by treatment, with residual HRP2: PCR negative and HS-RDT positive; (3) malaria-infected individuals without residual HPR2: PCR positive and HS-RDT negative test result; and, (4) non-infected individuals: both negative PCR and HS-RDT. The map of the study sites was generated using Quantum (Q)GIS version 7.5.0 with GRASS 7.4.4 programs. The administrative base map of The Gambia was created by downloading the “shapefiles” of The Gambia into QGIS and the administrative layers were generated. A final “shapefile” was created using the village coordinates and PCR infection prevalence and then layered onto the administrative map of The Gambia.

### Mathematical modelling

An existing deterministic age-structured dynamic model of malaria transmission was used to estimate the impact of MTAT with HS-RDT (compared to no intervention) on malaria prevalence and the proportion of clinical malaria cases averted [25]. The model assumes newborns start as susceptible with some maternally acquired immunity, which decays within 6 months. After an infectious mosquito bite, individuals have a probability of developing symptomatic or asymptomatic malaria. The probability of becoming infected after an infectious bite depends on the individual’s level of pre-erythrocytic immunity, and the probability of developing symptomatic or asymptomatic infection depends on their level of blood-stage immunity. The acquisition of both types of immunity depends on population-level of exposure. Individuals developing symptomatic malaria have a probability of being treated and then prophylactically protected. Untreated individuals with symptomatic infection are assumed to progress to asymptomatic infection after an average of 5 days. Asymptomatic individuals are assumed to remain *P. falciparum* infected for 310 days on average [26]. Infections are defined as either patent (detected by microscopy) or sub-patent (detected only by molecular method). Unless prophylactically protected by an anti-malarial drug, individuals can be re-infected or super-infected at any time.

The model was extended to include MTAT with HS-RDTs compared to either MTAT with conventional RDTs or MDA implemented as three monthly rounds per year for 2 consecutive years. The MTAT is implemented in the model whereby, at defined time points, a proportion of individuals (the intervention coverage) are tested. A proportion of infected individuals are assumed to test positive (determined by the diagnostic test’s sensitivity), treated and moved to the ‘prophylactic protection’ compartment. It is assumed that there is non-random coverage of who partakes in each intervention round: the population is split in two groups, one which partakes in all three treatment rounds, and one which has random and uncorrelated coverage over the three rounds.

The HS-RDT’s specificity is accounted for by assuming that a proportion of uninfected individuals will be HS-RDT positive and will receive treatment and prophylactic protection. MDA is implemented in the model by assuming that at defined time points, a proportion (equal to the MDA coverage) of individuals are treated (regardless of infection status) and move to the ‘prophylactic protection’ compartment. Coverage between treatment rounds is assumed to be correlated as described above.

The model used the HS-RDT sensitivity and specificity values estimated by the survey. It was assumed that sensitivity of conventional RDT is 22% lower than the HS-RDT and has 95% specificity [22]. The model assumed recently treated individuals remain RDT or HS-RDT positive for 15 days [27] and 30 days [28], respectively. The probability of detection during this period is equal to the sensitivity of each test in asymptomatic individuals. In the simulations, dihydroartemisinin–piperaquine (DHA–PQ) was used for MTAT and MDA with the post-treatment prophylactic period assumed to be 30 days on average. Finally, seven scenarios are simulated: (i) no mass intervention; (ii) MTAT with conventional RDT at 65%; (iii) 85% coverage; (iv) MTAT with HS-RDT at 65% coverage; (v) 85% coverage; (vi) MDA at 65% coverage, and (vii) 85% coverage. All interventions are implemented by three monthly round per year for 2 consecutive years, starting at the end of the dry season. All simulations are run according to different transmission intensity as determined by PCR-determined malaria prevalence namely; very low (5%), low (15%), moderate (30%), and high (60%).

### Consent

Community verbal approval was followed by individual written consent for adults who also provided consent for children below 18 years of age. Children between 12 and 17 years provided assent. Ethical approval was obtained from the Gambia Government/Medical Research Council Joint Ethics Committee.

## Results

A total of 4060 participants distributed in 47 villages were screened (Fig. 1); the median age was 13 years (IQR: 6, 31); 55.7% (2258/4054) of study participants were female and coverage of control interventions (LLIN and IRS) was high (Table 1).

**Table 1:** Participants demographic characteristics

Variable	N (%)
Age group (N = 4024)	
0 to < 5	667 (16.6)
5 to < 10	871 (21.7)
10 to < 20	996 (24.8)
20 to < 40	827 (20.6)
40 to < 90	663 (16.5)
Gender (N = 4054)	
Female	2258 (55.7)
Male	1796 (44.3)
Reported LLIN ownership (N = 3998)	
Yes	3799 (95.0)
Used LLIN the previous night (N = 4009)	
Yes	3512 (87.6)
Sprayed IRS in the previous 6 months (N = 3992)	
Yes	3642 (91.2)

The prevalence of *P. falciparum* infection was 15.0% (570/3798; 95% CI 13.9–16.1) by HS-RDT and 13.1% (497/3798; 95% CI 12.0–14.2) by PCR. HS-RDT’s sensitivity and specificity were 38.4% (191/497; 95% CI 34.2–42.7) and 88.5% (2922/3301; 95% CI 87.4–89.6), respectively (Table 2).

**Table 2:** Performance of highly sensitive rapid diagnostic tests as compared to PCR

PCR	HS-RDT		Total
	HS-RDT positive	HS-RDT negative	
Positive	191	306	497
Negative	379	2292	3301
Total	570	3228	3798

	Value (%)	95% CI	
Prevalence by HS-RDT	15.0	13.9–16.1	
Sensitivity	38.4	34.2–42.7	
Specificity	88.5	87.4–89.6	
Positive predictive value	33.5	29.6–37.4	
Negative predictive value	90.5	89.5–91.5	

Among individuals with fever (temperature ≥ 37.5 °C) or history of fever, malaria prevalence by HS-RDT was 17.1% (200/1172; 95% CI 14.9–19.2) and 14.1% (369/2610; 95% CI 12.8–15.5) in those without fever. HS-RDT sensitivity was similar in individuals with (41.5%, 63/152; 95% CI 33.6–49.3%) or without (37.3%, 128/343; 95% CI 32.2–42.4) fever or history of fever in the previous 24 h (p = 0.38).

**Fig. 2:**
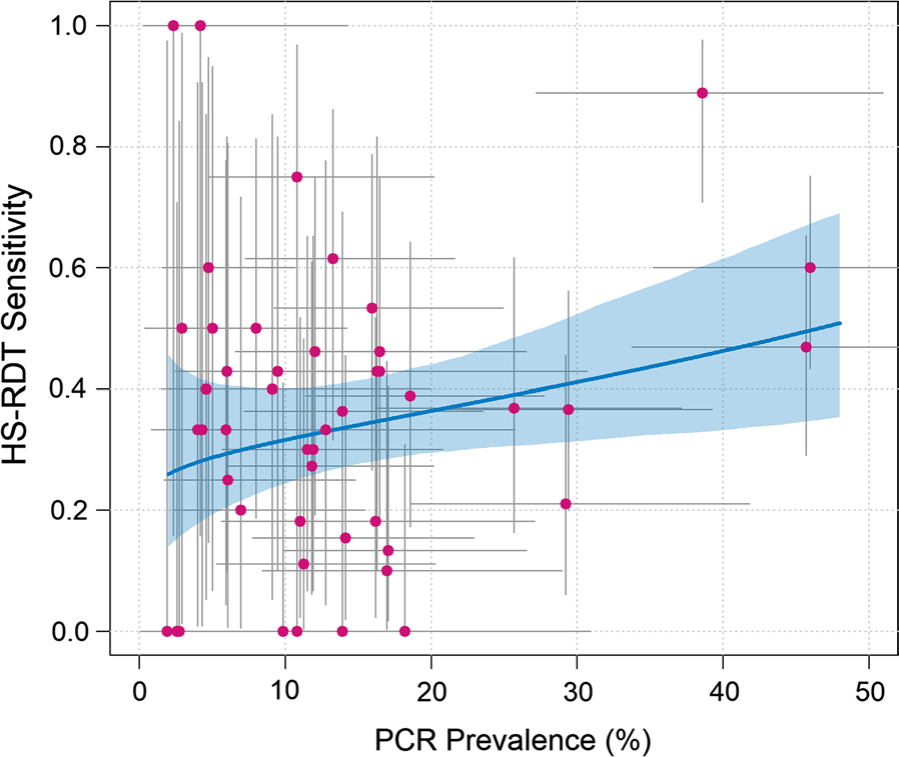
Association between the highly sensitive rapid diagnostic test sensitivity and *Plasmodium falciparum* infection prevalence by PCR. Filled pink circle: each pink dot represents a village and the grey horizontal and vertical lines are 95% confidence intervals associated with the prevalence and sensitivity estimates, blue solid line: the fitted relationship between the two quantities derived from a log-odds regression model between PCR and HS-RDT prevalence implemented within a Bayesian framework. Full details of model structure have been previously published [25]

**Fig. 3:**
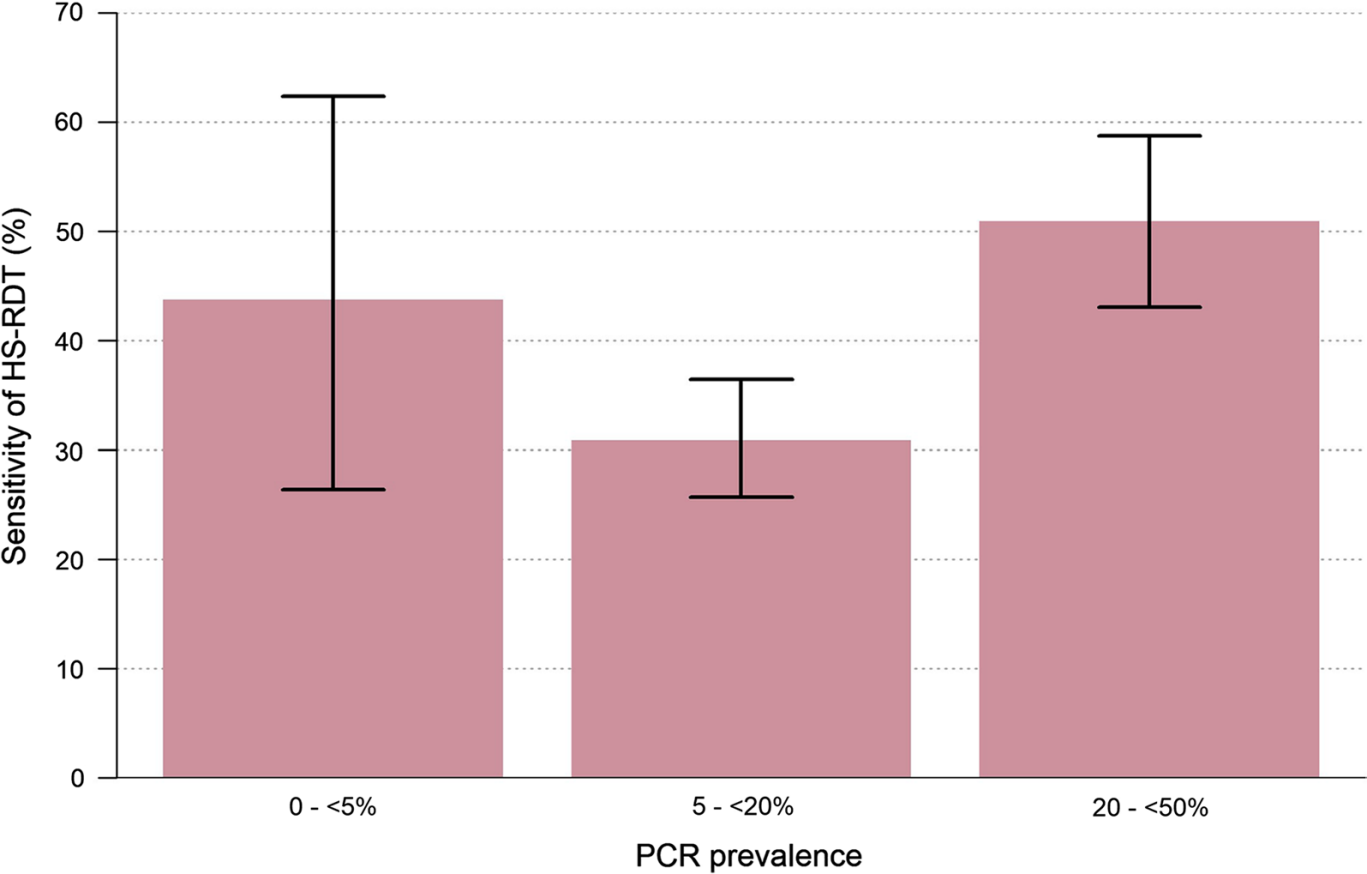
Performance of highly sensitive rapid diagnostic test by transmission intensity

The HS-RDT sensitivity increased with increasing *P. falciparum* prevalence by PCR (Fig. 2), and was the highest, 50.9% (85/167; 95% CI 43.3–58.5) in high transmission villages (20–50% prevalence) (Fig. 3). The HS-RDT sensitivity was significantly lower and specificity higher in villages with low-moderate prevalence than in those with high prevalence (p < 0.01) (Additional file 1: Table S1).

The HS-RDT specificity was significantly lower in high 76.1% (229/301, 95% CI 71.3–80.9) than in low-moderate transmission 89.3% (1916/2146, 95% CI 87.9–90.6) (χ^2^ = 42.5, p < 0.01) or very low transmission 90.9% (777/854, 95% CI 89.1–92.9) (χ^2^ = 43.9, p < 0.01) villages (Table 3). Additional file 1: Table S1 shows the comparison of the HS-RDT sensitivity and specificity by intensity of transmission.

**Table 3:** Performance of the highly sensitive rapid diagnostic test by malaria prevalence

	Very low transmission (prevalence < 5%)			Low-moderate transmission (prevalence 5 to < 20%)			High transmission (prevalence 20–50%)		
	HS-RDT			HS-RDT			HS-RDT		
	Positive	Negative	Total	Positive	Negative	Total	Positive	Negative	Total
PCR positive	14	18	32	92	206	298	85	82	167
PCR negative	77	777	854	230	1916	2146	72	229	301
Total	91	795	886	322	2122	2444	157	311	468
PCR prevalence	3.6 (2.5–5.1)			12.2 (10.9–13.5)			35.7 (31.3–40.2)		
Sensitivity (95% CI)	43.8 (26.4–62.3)			30.9 (25.6–36.1)			50.9 (43.3–58.5)		
Specificity (95% CI)	90.9 (89.1–92.9)			89.3 (87.9–90.6)			76.1 (71.3–80.9)		
Positive predictive value (95% CI)	15.4 (8.7–24.5)			28.6 (23.6–33.5)			54.1 (46.4–61.9)		
Negative predictive value (95% CI)	97.7 (96.4–98.7)			90.3 (89.0–91.6)			73.6 (68.7–78.5)		

**Fig. 4:**
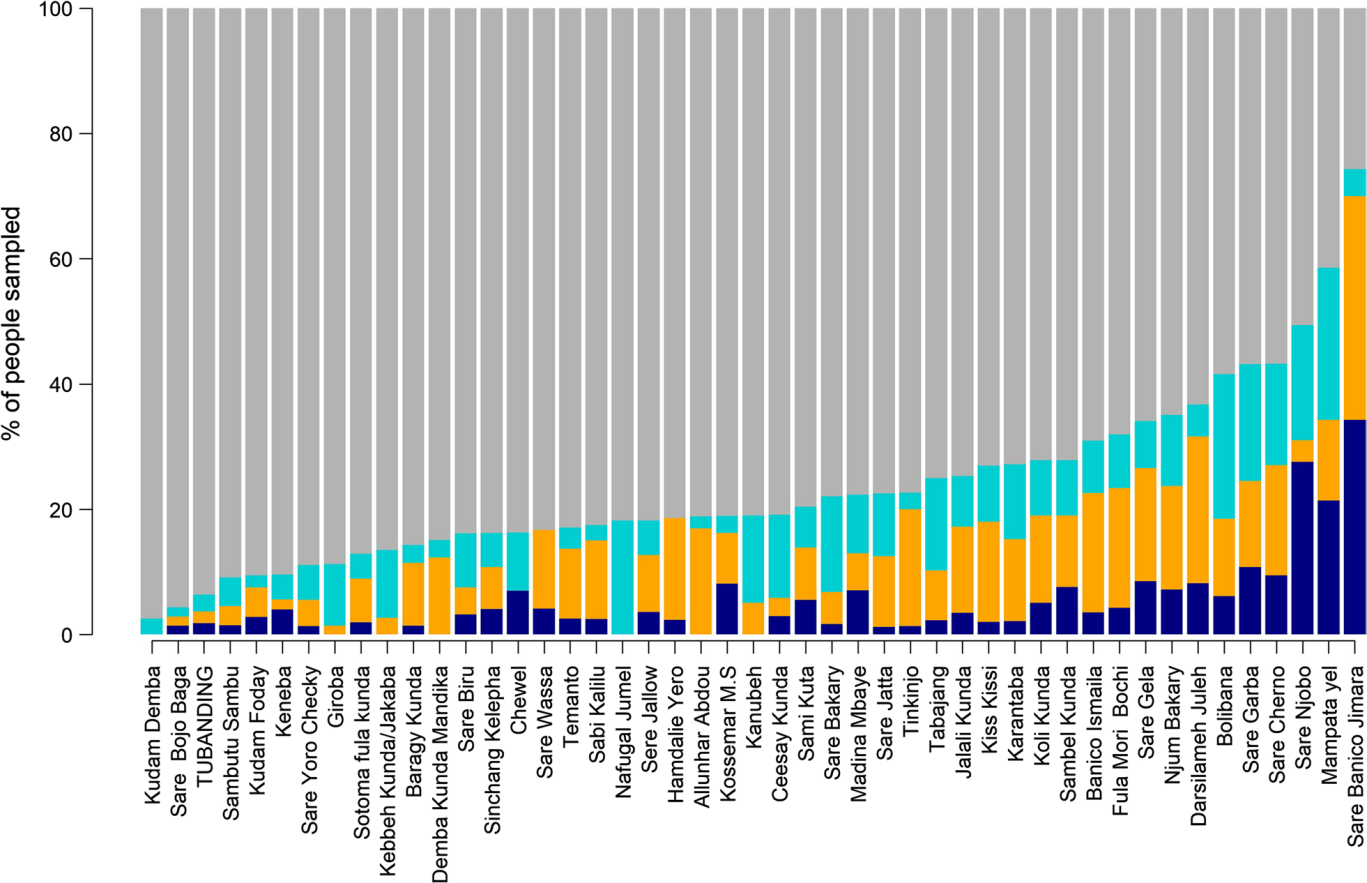
Prevalence of *Plasmodium falciparum* infections as determined by highly sensitive rapid diagnostic test and PCR by village. Dark blue bar: proportions of PCR positive HS-RDT positive infections, dark yellow bar: proportions of PCR negative HS-RDT positive infections, light blue bar: proportion of PCR positive HS-RDT negative infections, grey bar: proportions of PCR negative HS-RDT negative infections

More than half of PCR positive samples were negative by HS-RDT (306/497; 61.6%, 95% CI 57.3–65.8), with the largest proportion of ‘false negatives’ in villages with low-moderate transmission. The odds of being false negative in the low-moderate and high prevalence villages was not different than in the very low prevalence villages. (Additional file 2: Table S2, Fig. 4).

**Fig. 5:**
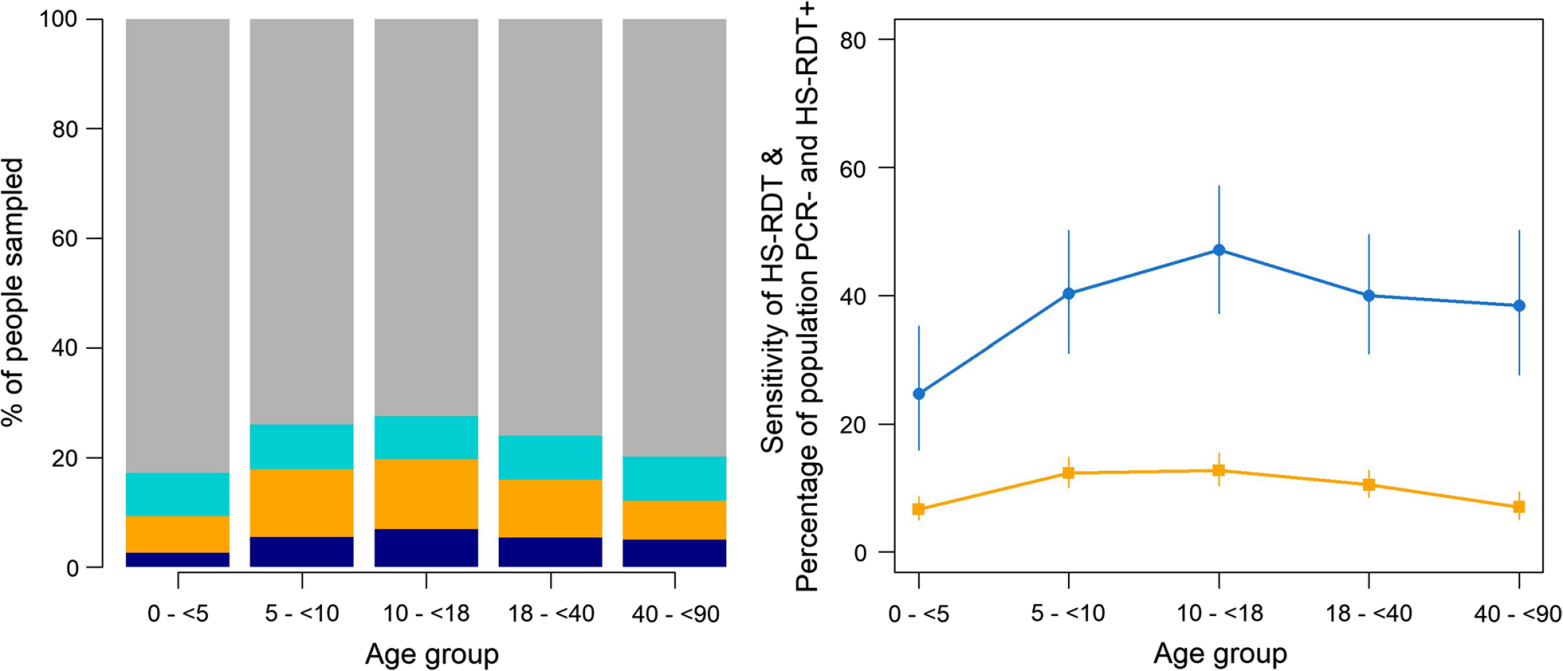
Performance of highly sensitive rapid diagnostic test by age. **a** Dark blue bar: percentage of population that are PCR positive HS-RDT positive, dark yellow bar: percentage of population that are PCR negative HS-RDT positive, light blue bar: percentage of population that are PCR positive HS-RDT negative, grey bar: percentage of population that are PCR negative HS-RDT negative. **b** Solid blue line: sensitivity of HS-RDT (compared to PCR) for different age groups, with 95% confidence intervals, solid red line: percentage of the population that are PCR negative and HS-RDT positive for different age groups, with 95% confidence intervals

The HS-RDT sensitivity did not differ between older children and adults but was significantly lower in children below 5 years than in older children and adults (Table 4, Fig. 5). The older children and adults had about 70% lower odds of being false negative, i.e., PCR positive and HS-RDT negative, than younger children (Table 5).

**Table 4:** Performance of highly sensitive rapid diagnostic test by age

	≤5 years	5 to < 10 years	10 to < 18 years	18 to < 40 years	40–90 years
	%, n/N (95% CI)	%, n/N (95% CI)	%, n/N (95% CI)	%, n/N (95% CI)	%, n/N (95% CI)
PCR prevalence	10.7%, 67/626 (8.3–13.1)	12.7%, 105/824 (10.5–15.2)	14.5%, 119/823 (12.1–16.9)	13.8%, 121/874 (11.6–16.1)	13.1, 81/619 (10.4–15.7)
Sensitivity	17.9%, 12/67 (8.7–27.1)	43.8%, 46/105 (34.3–53.3)	43.7%, 52/119 (34.8–52.6)	40.5%, 49/121 (31.7–49.2)	39.5%, 32/81 (28.9–50.2)
Specificity	93.0%, 520/5599 (90–95.1)	87.1%, 626/719 (84.6–89.5)	84.5%, 597/704 (81.8–87.6)	87.9%, 662/753 (85.6–90.3)	91.8%, 494/538 (89.5–94.1)
Positive predictive value	23.5%, 12/51 (11.9–35.2)	33.1%, 46/139 (25.3–40.9)	32.7%, 52/159 (25.4–39.9)	35.0%, 49/140 (27.1–42.9)	42.1%, 32/76 (31.0–53.2)
Negative predictive value	90.4%, 520/575 (88.03–92.8)	91.4%, 626/685 (89.3–93.4)	89.9%, 597/664 (87.6–92.2)	90.2%, 662/734 (88.03–92.3)	90.9%, 494/543 (88.6–93.4)
Comparison of HS-RDT sensitivity of older children and adults to children < 5 years	1	χ^2^ = 12.3, p < 0.01	χ^2^ = 12.6, p < 0.01	χ^2^ = 10, p < 0.01	χ^2^ = 8.2, p < 0.01

**Table 5:** Risk of being false negative: PCR positive and HS-RDT negative by age, LLIN use and gender

	HS-RDT false negatives n/N, (%)	OR (95% CI)	p value	AOR (95% CI)	p value
Age (years)					
≤ 5	55/67 (82.1)	1		1	
5 to < 10	59/105 (56.2)	0.28 (0.13–0.58)	< 0.01	0.28 (0.13–0.6)	< 0.01
10 to < 18	67/119 (56.3)	0.28 (0.14–0.6)	< 0.01	0.29 (0.14–0.6)	< 0.01
18 to < 40	72/121 (59.5)	0.32 (0.16–0.66)	< 0.01	0.32 (0.16–0.7)	< 0.01
40 to < 90	49/81 (60.5)	0.33 (0.16–0.7)	< 0.01	0.33 (0.15–0.7)	< 0.01
IRS					
No		1			
Yes		0.52 (0.3–1.03)	0.06	0.50 (0.3–1.0)	0.05
LLIN at night					
No		1			
Yes		0.89 (0.5–1.6)	0.69		
Gender					
Male		1			
Female		0.89 (0.6–1.3)	0.53		

Overall, 11.5% (379/3301) individuals were ‘false positives’, i.e. HS-RDT positive and PCR negative. This proportion increased with increasing malaria prevalence by PCR with the odds of being false positive significantly higher in the high transmission villages than in very low transmission villages (Additional file 3: Table S3, Fig. 4). Only 3.7% (11/299; 95% CI 1.6–5.8) false positive participants reported taking an anti-malarial treatment within the previous month, with similar proportions in low-moderate (4.8%, 8/78) and high (4.2%, 3/72) transmission villages.

**Fig. 6:**
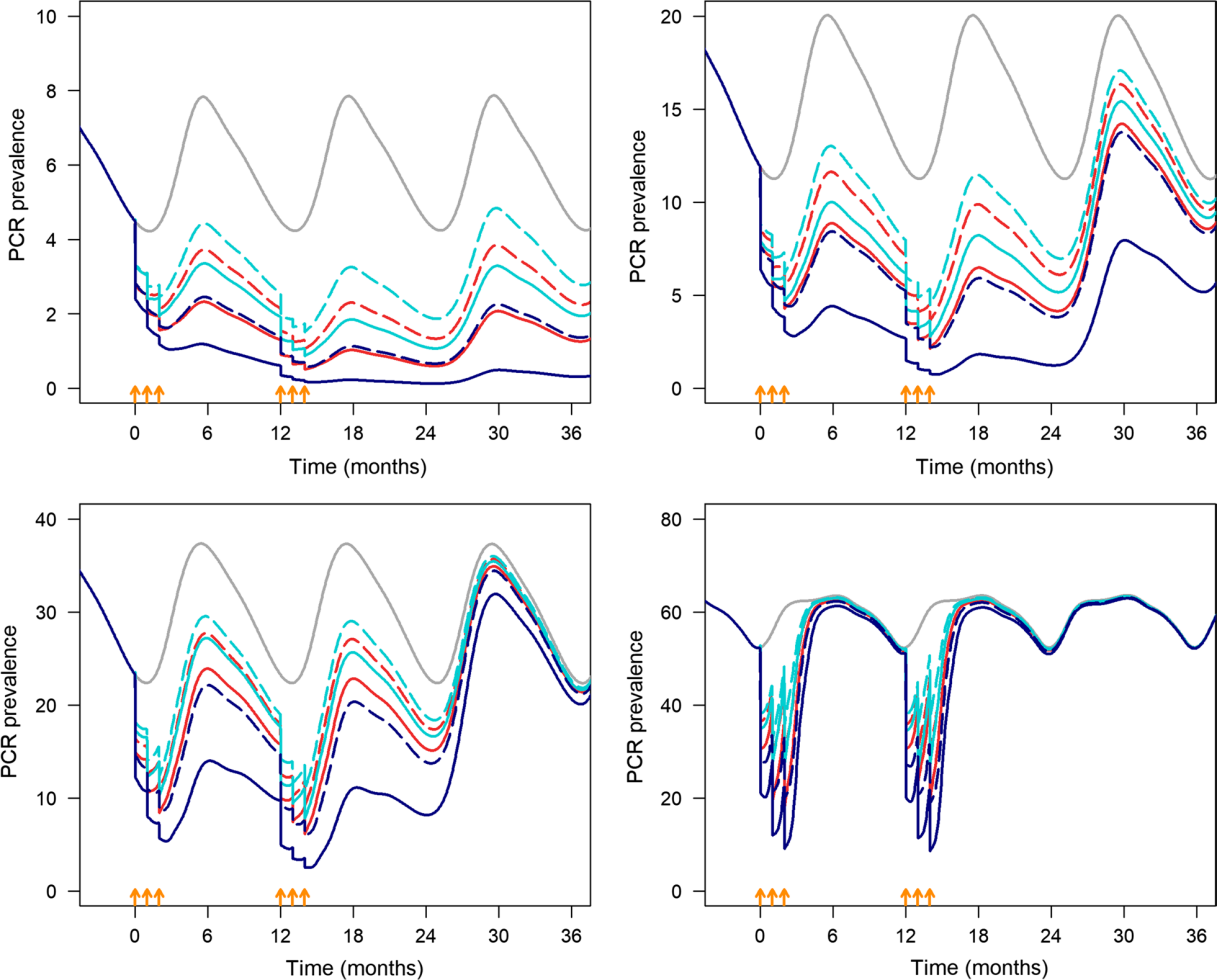
Mathematical model simulation of the predicted impact of the interventions (MTAT with HS-RDT, MTAT with conventional RDT, and MDA) implemented for three monthly rounds for 2 consecutive years on PCR prevalence. The intervention is implemented in a seasonal transmission setting with four levels of transmission intensity. The orange arrows indicate the timing of each MTAT/MDA round. Solid grey line: no MTAT, dashed light blue line: co-RDT MTAT, 65% coverage, solid light blue line: co-RDT MTAT, 85% coverage, dashed red line: HS-RDT MTAT, 65% coverage, solid red line: HS-RDT MTAT, 85% coverage, dashed dark blue line: MDA, 65% coverage, dashed solid dark blue line: MDA, 85% coverage

For the malaria transmission model, the HS-RDT sensitivity is assumed to be 38.4%. The proportion of false positives (PCR negative and HS-RDT positive) was assumed to be 10%. At very low prevalence (~ 5%), three monthly rounds of MTAT with HS-RDT per year at 85% coverage would decrease prevalence by 80%, from 5 to around 1%, up to 18 months after the final MTAT round (solid red line, Fig. 6a). In addition, 78.4% of clinical cases would be averted at the end of the 2 years. At 65% coverage malaria prevalence would decrease to 2% in 2 years averting 62.0% of malaria cases but it would return to pre-MTAT levels 12 months after the last monthly round of MTAT. The impact of MTAT with conventional RDT would be lower; at 85 and 65% coverage, it would avert 66.5 and 51.8% of cases, respectively. The effect of the intervention would be more marked for MDA as 93 and 80.5% of cases would be averted at 85 and 65% coverage, respectively (Additional file 4: Table S4 and Fig. 6a).

At low prevalence (~ 15%), three monthly rounds of MTAT with HS-RDT at 85% coverage would decrease prevalence to around 7 and 5% in the first and second year, respectively. Prevalence would return to almost pre-MTAT levels 12 months after the last round if the intervention is not continued (solid red line, Fig. 6b); the intervention would avert 66.8% of malaria cases over 2 years (Additional file 4: Table S4). MDA at 85% coverage would decrease malaria prevalence from 12 to about 5% in the first year and 3% in the second year that would increase only after 18 months.

In moderate (prevalence ~ 30%) and high (prevalence ~ 60%) transmission areas, MTAT with HS-RDT at 85% coverage would decrease malaria prevalence by about 60% during the intervention months; however, prevalence would rapidly return to pre-MTAT levels after the last round, i.e. 6 months in moderate and 3 months in high transmission areas (Fig. 6c, d). In moderate transmission settings, MTAT with a HS-RDT over 2 years would avert 53 and 40% of malaria cases at 85 and 65% coverage, respectively (Additional file 4: Table S4). The percentage of cases averted is lower for MTAT with a conventional RDT (42.8 and 33.4% at 85 and 65% coverage, respectively) and higher for MDA (80.6 and 61.9% at 85 and 65% coverage, respectively). In all transmission settings, 85% coverage of MTAT with a HS-RDT had similar impact as MDA with a coverage of 65%.

## Discussion

HS-RDT sensitivity was low, missing 62% of infected individuals identified by PCR, but was similar to that reported from Myanmar (36.6%) [18], Papua New Guinea (51%) [19] and Ethiopia (33.9%) [29], confirming the poor performance of this diagnostic test in detecting low-density malaria infections. Despite this, malaria prevalence estimated by HS-RDT was similar, even slightly higher, to that determined by PCR, a result partly due to the high proportion of false positives, i.e., samples positive by HS-RDT and negative by PCR. The latter could be explained by residual circulating malaria antigen (HRP2) lasting up to 28 days after infection clearance [28, 30], from either anti-malarial treatment [31] or by the immune system [32]. However, some individuals may have been infected with low parasite density fluctuating below the detection threshold of PCR [32–34]. This could be investigated using a larger volume of blood for the molecular analysis [19]. In these individuals with low-density infections, parasite density may increase and are detectable by RDTs or microscopy [35].

Most discrepant results were represented by false negatives, i.e., negative by HS-RDT and positive by PCR, possibly due to the lower detection threshold of PCR compared to HS-RDT [23]. False negative individuals may harbour low-density infections but they may still be infectious to the vector or may progress towards clinical disease [33, 35]. In addition, a false negative result could be due to the deletion of the HRP2 gene [36], HRP2 antigens [11] although this maybe unlikely in this setting. As *P. falciparum* density was not measured, it is not possible to determine whether false negative samples had parasite densities below the HS-RDT detection threshold. Such limitation also prevents to establish whether the differential performance of the HS-RDT by age group and prevalence could be explained by variations in parasite densities. Indeed, HS-RDT’s sensitivity and specificity varied by malaria prevalence, with the highest sensitivity and the lowest specificity in high transmission villages. This may be due to the higher probability of being infected (and re-infected) at any given moment (high sensitivity), possibly with higher parasite densities in individuals repeatedly infected, and by the detection of circulating *P. falciparum* HRP2 antigens from past infections (low specificity). Where malaria prevalence was very low, despite missing more than half of infected individuals, HS-RDT would identify those with circulating parasite antigens who had been infected about 1 month earlier [37, 38], indicating areas of ongoing transmission.

In this context, treating HS-RDT positive individuals, either with a detectable infection by PCR or with circulating HRP2 from a previous infection, could further decrease malaria prevalence, when using a treatment with a long post-treatment prophylactic period such as dihydroartemisinin–piperaquine (DHA–PQ) [39]. When malaria transmission significantly decreases, it becomes heterogenous and focal, with higher transmission in a few compounds or households and lower transmission in surrounding households [40]. In this context, infected individuals or with evidence of a previous infection may indicate areas of ongoing transmission. This is plausible in The Gambia where households with a malaria-infected individual at the beginning of the transmission season were more likely to have one household member acquire clinical malaria [6].

Trials evaluating the effect of multiple MTAT using conventional RDTs found temporary reductions of malaria prevalence in communities in Burkina Faso and Zambia [14, 41], and in schools in Kenya [16]. Such transient effect is probably due to the inability of conventional RDTs to identify and treat individuals with low-density infections. In addition, according to a published mathematical model, MTAT with sensitive tests would increase the probability of local malaria elimination only in very low transmission settings [42]. The mathematical model presented in this paper compared MDA and MTAT with either conventional RDT or HS-RDT. In all transmission scenarios, MDA would be more efficacious than MTAT in reducing malaria prevalence, although such effect would be transient in areas of intense transmission.

Nevertheless, at very low or low transmission intensities, MTAT with HS-RDT at 85% coverage would have a similar effect than MDA at 65% coverage and reduce malaria prevalence by 80%, from 5 to 1% for about 18 months after the last MTAT round. At lower coverage, MTAT with HS-RDT would have a much lower effect, like that obtained by MTAT with conventional RDT at 85% coverage. The success of MDA depends on high coverage which in turn is heavily influenced by its acceptability by the local populations, which can change and decrease over time. MTAT may be more acceptable to the local population as it identifies and treats infected individuals [43, 44]. An additional advantage would be to reduce the risk of treatment-related adverse events as only HS-RDT positive individuals, in this context a small percentage of the whole population, would be treated. Moreover, MTAT with a HS-RDT may be an alternative to MDA is areas of multi-drug resistance [45] as it would reduce the drug pressure and thus the selection of resistant parasites.

The timing of MTAT implementation may also be important; in a highly seasonal setting, the three rounds should be implemented at the end the dry season, before the malaria seasonal transmission starts. This may improve the HS-RDT performance as individuals infected during the previous transmission season would have cleared any residual parasite-circulating antigen, thus reducing the percentage of false positives.

The mathematical model used to estimate the effect on MTAT with HS-RDT is based on several assumptions. These predictions would need to be empirically confirmed by field studies, e.g., cluster randomized trials, which may compare MDA against MTAT or, depending on the malaria prevalence, the two interventions implemented sequentially, i.e., MDA to reduce malaria prevalence to levels at which MTAT could have an additional effect.

## Conclusions

HS-RDTs were developed with the aim of reducing the human reservoir of infection as they would identify asymptomatic, malaria-infected individuals [46] to be treated with an artemisinin-based combination treatment. Despite the sub-optimal sensitivity and specificity, HS-RDT could be used to identify groups of individuals at higher risk of infection. The mathematical model predicts that in low to very low transmission areas, MTAT with HS-RDT would substantially decrease malaria prevalence. Such prediction would need to be confirmed by cluster-randomized trials.

## Supplementary information

**Additional file 1: Table S1.** Comparison of HS-RDT sensitivity and specificity by intensity of transmission.

**Additional file 2: Table S2.** Risk of being false negative: PCR positive and HS-RDT negative by malaria prevalence.

**Additional file 3: Table S3.** Risk of false positive: PCR negative and HS-RDT positive by transmission intensity.

**Additional file 4: Table S4.** Percentage reduction in clinical malaria cases over 2 years after simulated MTAT and MDA scenarios shown in Fig. 6.

## Data Availability

The participant data has been collected following provision of informed consent under the prerequisite of strict participant confidentiality (SCC reference 1318). Access to participant data can be accessed following review of the request by the Gambia Government/Medical Research Council Joint Ethics Committee to protect the rights and interests of the study participants. The review process and release of data will be facilitated by Medical Research Council and access will not be unduly restricted. The MRC unit The Gambia at LSHTM is committed to data sharing and open access via its respective policies. Contact information for the Gambia Government/MRC Joint Ethics Committee: Ms Naffie Jobe, Secretary Gambia Government/MRCG at LSHTM Joint Ethics Committee, njobe@mrc.gm.

## Abbreviations

DHA–PQ: dihydroartemisinin–piperaquine
DNA: deoxyribonucleic acid
ELISA: enzyme-linked immunosorbent assay
HDSS: health and demographic surveillance system
HS-RDT: highly sensitive rapid diagnostic test
HRP2: histidine rich protein 2
IRS: indoor residual spraying
LLIN: long-lasting insecticide net
REDCap: research electronic data capture
MDA: mass drug administration
MTAT: mass testing and treatment
PCR: polymerase chain reaction
QGIS: quantum geographic information system
(q)RT-PCR: quantitative reverse-transcription polymerase chain reaction
*var*ATS: *var* gene acidic terminal sequence
