# Supplementary material for: Field performance of the malaria highly sensitive rapid diagnostic test in a setting of varying malaria transmission
Source: Malar J. 2019 Aug 27;18:288. doi: 10.1186/s12936-019-2929-1 (PMC6712604; doi:10.1186/s12936-019-2929-1)
Supplement: Supplementary file 1 — Additional file 1: Table S1. Comparison of HS-RDT sensitivity and specificity by intensity of transmission. [file 12936_2019_2929_MOESM1_ESM.docx]

### **Table S1 Comparison of HS-RDT sensitivity and specificity by intensity of transmission**

| Sensitivity |  |  |
| --- | --- | --- |
|  | Low-moderate transmission (prevalence 5- ≤20%) | High transmission  (prevalence 20-50%) |
|  | (30.9%, 92/298) | (50.9, 85/167) |
| Very low transmission (43.8%, 14/32) | χ^2^=2.2, P=0.14 | χ^2^=0.6, P=0.46 |
| Low-moderate transmission | - | χ^2^=18.2, P ≤ 0.01 |
| Specificity |  |  |
|  | Low-moderate transmission | High transmission |
|  | (89.3%, 1,916/2,146) | (76.1%, 229/301) |
| Very low transmission (90.9%, 777/854) | χ^2^=1.9, P=0.2 | χ^2^=43.9, P<0.01 |
| Low-moderate transmission | - | χ^2^=42.5, P<0.01 |
