# Supplementary material for: Field performance of the malaria highly sensitive rapid diagnostic test in a setting of varying malaria transmission
Source: Malar J. 2019 Aug 27;18:288. doi: 10.1186/s12936-019-2929-1 (PMC6712604; doi:10.1186/s12936-019-2929-1)
Supplement: Supplementary file 2 — Additional file 2: Table S2. Risk of being false negative: PCR positive and HS-RDT negative by malaria prevalence. [file 12936_2019_2929_MOESM2_ESM.doc]

### **Table S**2 Risk of being false negative: PCR positive and HS-RDT negative by malaria prevalence

| **Intensity of transmission** | **HS-RDT false negative**  **n/N, % (95% CI)** | **OR**  **(95% CI)** | **P value** | **AOR**  **(95% CI)** | **P value** |
| --- | --- | --- | --- | --- | --- |
| Very low: prevalence <5% | 56.3%, 18/32  (39.1-73.4) | 1 |  |  |  |
| Low-moderate: prevalence  5-<20% | 69.1%, 206/298  (63.9-74.4) | 1.7  (0.8-3.7) | 0.14 | 1.9  (0.9-4.0) | 0.09 |
| High: prevalence 20-50% | 49.1%, 82/167  (41.5-56.7) | 0.75  (0.4-1.6) | 0.46 | 0.82  (0.4-1.8) | 0.62 |
