# Supplementary material for: Field performance of the malaria highly sensitive rapid diagnostic test in a setting of varying malaria transmission
Source: Malar J. 2019 Aug 27;18:288. doi: 10.1186/s12936-019-2929-1 (PMC6712604; doi:10.1186/s12936-019-2929-1)
Supplement: Supplementary file 3 — Additional file 3: Table S3. Risk of false positive: PCR negative and HS-RDT positive by transmission intensity. [file 12936_2019_2929_MOESM3_ESM.docx]

### Table S3 Risk of false positive: PCR negative and HS-RDT positive by transmission intensity

| **Transmission intensity** | **HS-RDT false positives: n/N, % (95% CI)** | **OR**  **(95% CI)** | **p value** | **AOR**  **95% CI** | **P value** |
| --- | --- | --- | --- | --- | --- |
| Very low: prevalence <5% | 77/854, 9.0%,  (7.09-10.9) | 1 |  | 1 |  |
| Low-moderate: prevalence  5-<20% | 230/2,146, 10.7%  (9.4-12.0) | 1.2  (0.9-1.6) | 0.17 | 1.2  (0.9-1.6) | 0.12 |
| High: prevalence  20-<50% | 72/301, 23.9%  (19.1-28.7) | 3.2  (2.2-4.5) | <0.01 | 3.3  (2.3-4.7) | <0.01 |
