# Supplementary material for: Field performance of the malaria highly sensitive rapid diagnostic test in a setting of varying malaria transmission
Source: Malar J. 2019 Aug 27;18:288. doi: 10.1186/s12936-019-2929-1 (PMC6712604; doi:10.1186/s12936-019-2929-1)
Supplement: Supplementary file 4 — Additional file 4: Table S4. Percentage reduction in clinical malaria cases over 2 years after simulated MTAT and MDA scenarios shown in Fig. 6. [file 12936_2019_2929_MOESM4_ESM.docx]

**Table S4 Percentage reduction in clinical malaria cases over two years after simulated MTAT and MDA scenarios shown in Fig. 6**

|  |  | Clinical malaria cases averted over two years (%) compared to no intervention | | |
| --- | --- | --- | --- | --- |
| Transmission intensity | Coverage | MTAT with conventional RDTs | MTAT with HS-RDT | MDA |
| Very low prevalence (~5%) | 85% | 66.5 | 78.4 | 93.0 |
| Very low prevalence (~5%) | 65% | 51.8 | 62.0 | 80.5 |
| Low prevalence (~15%) | 85% | 59.8 | 66.8 | 89.0 |
| Low prevalence (~15%) | 65% | 44.7 | 52.4 | 72.6 |
| Moderate prevalence (~30%) | 85% | 42.8 | 52.5 | 80.6 |
| Moderate prevalence (~30%) | 65% | 33.4 | 40.4 | 61.9 |
| High prevalence (~60%) | 85% | 24.1 | 32.9 | 48.9 |
| High prevalence (~60%) | 65% | 17.7 | 24.2 | 38.2 |
